# ENavigating the barriers to multi-regional research administrative data access: Forward thinking solutionsnada

**DOI:** 10.23889/ijpds.v9i5.2629

**Published:** 2024-09-18

**Authors:** Donna Curtis Maillet, Nicole Yada

**Affiliations:** 1 NB-IRDT University of New Brunswick; 2 University of Toronto

In 2021, aware of both the importance of pan-Canadian data access for advancing health research and the conditions and requirements that make administrative data access onerous, HDRN Canada worked with CATCO the Canadian arm of an international COVID-19 clinical trial to create a roadmap for navigating the barriers to multi-regional administrative data access in Canada.

While developing the roadmap, data access request processes were studied in real time for a multi-region study seeking access to link administrative data to clinical trial data, at the individual level. The intent was to move beyond anecdotal evidence and document the specific nature of policy and processes that hamper data access. The case study allowed the study team to define the barriers permitting application of tangible mitigating measures.

In 2022, barriers such as the lack of ability to share data across provincial borders became too great to continue the study. Although the roadmap was not completed, insights were gained for waypoints along the path enabling HDRN Canada to make important process improvement to harmonize data access across regions. Solutions to challenges with data access request forms, regional data flows, data sharing partner relationships, data sharing agreements, and REB and project reviews were identified.

Recently pan-Canadian initiatives have released important guidance for addressing data access challenges. The adoption of this guidance is paramount to ensure Canada continues to meaningfully contribute to health research.

Forward thinking solutions to data access challenges today will establish the necessary infrastructure for a future with minimal data access barriers.

